# Eye spectral sensitivity in fresh- and brackish-water populations of three glacial-relict *Mysis* species (Crustacea): physiology and genetics of differential tuning

**DOI:** 10.1007/s00359-016-1079-y

**Published:** 2016-03-16

**Authors:** Kristian Donner, Pavel Zak, Martta Viljanen, Magnus Lindström, Tatiana Feldman, Mikhail Ostrovsky

**Affiliations:** Department of Biosciences, University of Helsinki, P.O.Box 65 (Viikinkaari 1), 00014 Helsinki, Finland; Emanuel Institute of Biochemical Physics, Russian Academy of Sciences, Moscow, Russia; Tvärminne Zoological Station, University of Helsinki, Hanko, Finland; Department of Molecular Physiology, Biological Faculty, Lomonosov Moscow State University, Moscow, Russia

**Keywords:** Vision, Rhodopsin, Phenotypic plasticity, Evolutionary adaptation, Compound eye, Polarization sensitivity

## Abstract

Absorbance spectra of single rhabdoms were studied by microspectrophotometry (MSP) and spectral sensitivities of whole eyes by electroretinography (ERG) in three glacial-relict species of opossum shrimps (*Mysis*). Among eight populations from Fennoscandian fresh-water lakes (*L*) and seven populations from the brackish-water Baltic Sea (*S*), *L* spectra were systematically red-shifted by 20–30 nm compared with *S* spectra, save for one *L* and one *S* population. The difference holds across species and bears no consistent adaptive relation to the current light environments. In the most extensively studied *L*–*S* pair, two populations of *M. relicta* (*L*_p_ and *S*_p_) separated for less than 10,000 years, no differences translating into amino acid substitutions have been found in the opsin genes, and the chromophore of the visual pigments as analyzed by HPLC is pure A1. However, MSP experiments with spectrally selective bleaching show the presence of two rhodopsins (*λ*_max_ ≈ 525–530 nm, MWS, and 565–570 nm, LWS) expressed in different proportions. ERG recordings of responses to “red” and “blue” light linearly polarized at orthogonal angles indicate segregation of the pigments into different cells differing in polarization sensitivity. We propose that the pattern of development of LWS and MWS photoreceptors is governed by an ontogenetic switch responsive to some environmental signal(s) other than light that generally differ(s) between lakes and sea, and that this reaction norm is conserved from a common ancestor of all three species.

## Introduction

**Fig. 1:**
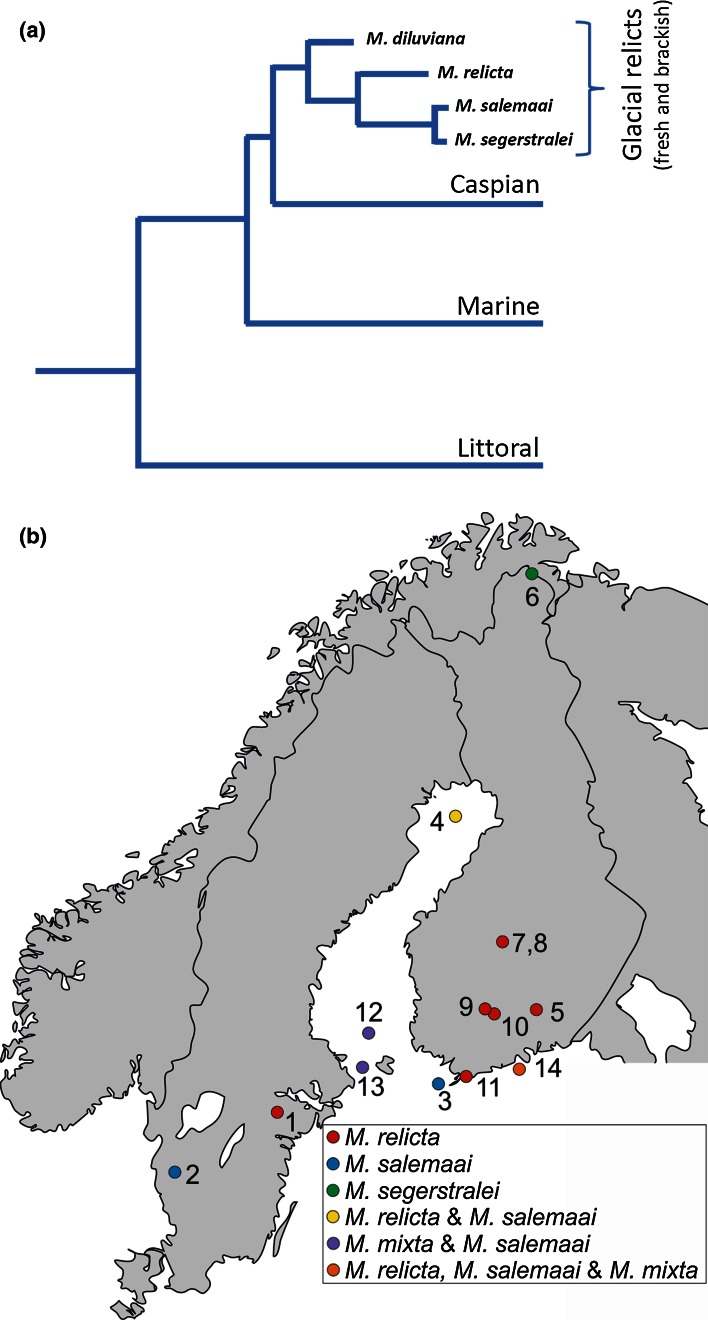
**a** Phylogeny of the glacial-relict *Mysis* species and their relation to other *Mysis* species flocks, based on multilocus sequence data and morphology (after Audzijonyte et al. 2005). Besides the glacial-relict species *M. relicta*, *M. salemaai* and *M. segerstralei*, the present study includes the species *M. mixta* as an outgroup representing the marine/Caspian clade. **b** Geographic locations of the populations included in the present study (see Table 1)

The genus *Mysis* (opossum shrimps) comprises a great number of species widely distributed over the arctic and temperate zones of the northern hemisphere. Among these are four glacial-relict sibling species inhabiting fresh and brackish waters, three in northern Europe (*M. relicta*, *M. salemaai* and *M. segerstralei*) and one in North America (*M. diluviana*) (Väinölä et al. 1994). Molecular evidence indicates that the evolutionary radiation of this group (formerly regarded as a single species *M. relicta*, *sensu lato*) dates back ≥2 Myr, although the divergence of *M. segerstralei* and *M. salemaai* is more recent (Väinölä 1986; Audzijonyte et al. 2005; Audzijonyte and Väinölä 2005, 2006). This as well as the relation of the whole group to the wider genus *Mysis* is schematically shown in Fig. 1a (cf. Audzijonyte et al. 2012). The “glacial-relict” branch of the primarily marine genus *Mysis* is so called because isolation patterns and speciation in northern coastal seas and lakes have been inextricably linked to Pleistocene glaciation history. The Fennoscandian populations have experienced repeated switches between marine, brackish/coastal/estuarine and freshwater conditions throughout the Pleistocene, up until their comparatively recent (<10 kyr, Eronen et al. 2001) postglacial isolation in inland lakes and the brackish-water Baltic Sea. Most likely, these switches have often been associated with significant changes in light conditions.

**Fig. 2:**
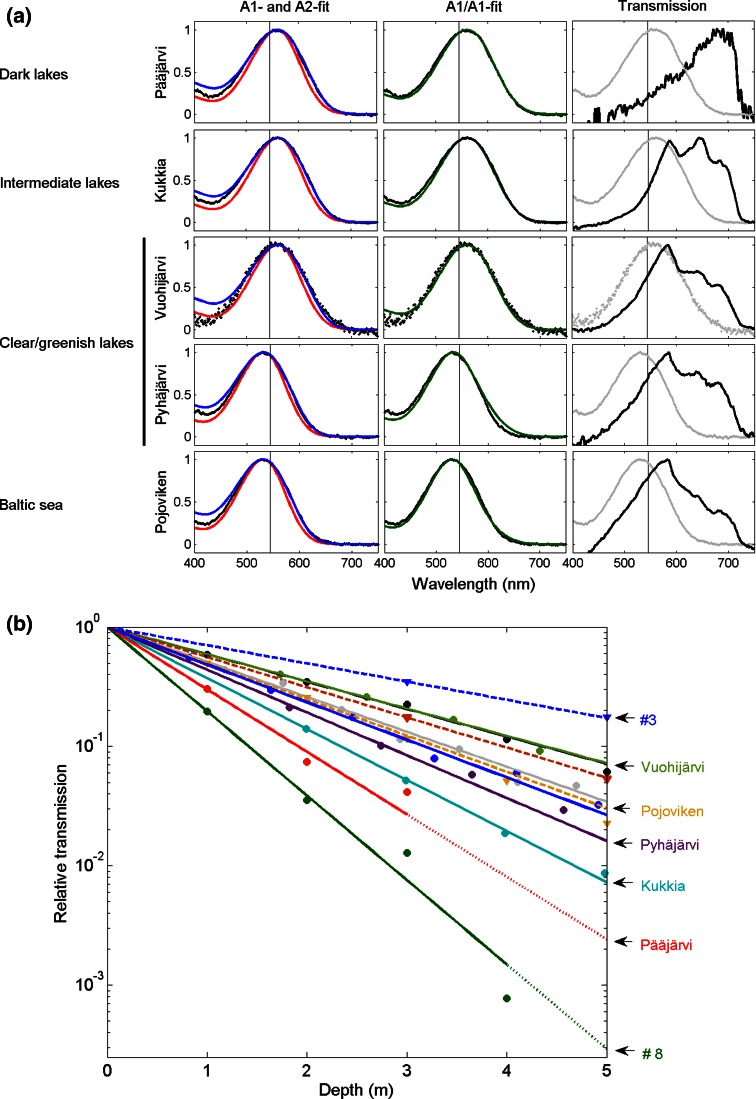
**a** Columns 1 and 2: absorbance spectra (*black*) of single *M. relicta* individuals from four lake populations (rows 1–4) and one sea population (row 5). Each spectrum is an average of recordings from ~10 to 20 single rhabdoms of one individual. The *smooth curves* in column 1 are GFRKD templates for A2 pigments fitted to the data (*blue*) and for A1 pigments locked to the same *λ*_max_ values (*red*). The *λ*_max_ values, determined from the A2 fits, are (from *top* to *bottom*): 559.5 nm (location/population #10 in Fig. 1b; Table 1), 560.3 nm (#9), 559.8 nm (#5), 534.3 nm (#7) and 531.0 nm (#11). In column 2 the same data have been fitted with linear sums of GFRKD templates for two A1 pigments (*P*_I_ and *P*_II_). Thus the curves are defined by three parameters [*λ*_max_(*P*_I_), *λ*_max_(*P*_II_), (*P*_I_:*P*_II_)]. The values *λ*_max_(*P*_LWS_) = 570 nm and *λ*_max_(*P*_MWS_) = 525 nm were assumed to be the same for all, as they represent the same species (*M. relicta*), whereby (*P*_LWS_:*P*_MWS_) = 0.76:0.24 (rows 1–3: locations #10, 9 and 5), 0.30:0.70 (row 4, location #7), and 0.21:0.79 (row 5; location #11). Column 3: the spectrum of downwelling light (*black*) at 1 m depth in the respective habitats (*black*). The recorded absorbance spectra from the two other columns have been included for comparison (*light gray*). In all panels, a *vertical line* has been drawn at 545 nm to facilitate distinction of “L-type” (*λ*_max_ > 545 nm) and “S-type” (*λ*_max_ < 540 nm) absorbance spectra (cf. Figure 3). **b** Light attenuation at the wavelength of maximal transmission (WMTL) as function of depth below surface in all the habitats where these measurements have been made (Table 1). *Dashed lines* and *triangles* refer to Baltic Sea habitats, *full-drawn lines* and *circles* to fresh-water habitats. Indicated by *arrows* are the five habitats of the samples in *panel* **a**, plus the clearest (location #3, Baltic Sea) and darkest of all (#8, Lake Mahlunjärvi). All functions have been normalized to 1 at the surface (i.e., 10° at depth 0) to remove effects of varying daylight intensity at the times of measurement. Extrapolations beyond the last points where measurements could be made in the two darkest habitats (Pääjärvi and #8) are marked by dotted lines

Concurrently, their vision has undergone apparently rapid changes in spectral sensitivity. The first report of what looked like ultra-fast evolutionary adaptation to different light conditions in *M. relicta* came from two populations living in different environments in southern Finland: the greenish coastal Pojoviken Bay and the dark brown Lake Pääjärvi (Lindström and Nilsson 1988; locations #11 and #10 in Fig. 1b). These populations will be denoted *S*_p_ (“Sea, Pojoviken”) and *L*_p_ (“Lake, Pääjärvi”). The ERG sensitivity spectrum of the *L*_p_ eye was found to be red-shifted by ca 30 nm compared with that of the *S*_p_ eye. This appeared to be clearly “adaptive” in a habitat where peak light transmission is at 680 nm and 99 % of all light is restricted to wavelengths >550 nm even a few meters below the surface (Lindström 2000; cf. Figs. 2, 7 of the present study). As the phylogenies, paleogeography and current habitats of mysid species and populations were more extensively mapped (Audzijonyte et al. 2005; Audzijonyte and Väinölä 2005), it became evident what a rich and unique model system they offer for studying changes in the spectral properties of vision (adaptive or not) on very different time scales.

Surprisingly, a similar 20–30 nm red shift of vision in fresh-water (“lake”, *L*) populations compared with brackish-water (“sea”, *S*) populations was found across all three European glacial-relict *Mysis* species, overriding ≥2 Myr of separate evolution and opsin gene divergence (Audzijonyte et al. 2012). The present article offers a review and reappraisal of results from more than a decade in the light of a large body of new data. Jokela-Määttä et al. (2005) concluded that the European glacial-relict *Mysis* species possess a single A2 visual pigment and that spectral differences must be due to differences in the (single) opsin. We now propose that all express two A1 visual pigments in different proportions under the control of an essentially bistable developmental switch responding to some environmental signal(s) that generally differ(s) between fresh and salt/brackish water. We hypothesize that such a genetic reaction norm (Woltereck 1909, 1928) conserved from a common ancestor underlies the dominant bimodal *L*–*S* distribution of spectral sensitivities across species, while evolutionary divergence of the opsins between species contributes comparatively minor variation components.

## Materials and methods

### Animals

Figure 1a shows the phylogenetic position within the genus *Mysis* of the three glacial-relict species *M. relicta*, *M. salemaai* and *M. segerstralei* chiefly considered in the present work. We include data on seven brackish-water (*S*) and eight fresh-water (*L*) populations from 14 locations as shown on the map in Fig. 1b. In addition, we have included three populations of *M. mixta* (from locations #12–14) belonging to the marine-Caspian clade.

The data on populations from locations #1–6 are from earlier studies by Jokela-Määttä et al. (2005) and Audzijonyte et al. (2012). For the new experiments, mysids were captured at locations #7–14 between October 2013 and August 2014 by horizontal netting from the top of the sediment (population #11) or by vertical net from the entire water column (all the others). After capture, the animals were transferred in light-tight cooler boxes to aquaria, where they were kept in darkness at +7 °C. When studying samples from locations where several mysid species coexist, we determined the species of each specimen morphologically (by microscopic inspection) and/or molecularly after the experiment (Väinölä et al. 2002; Audzijonyte and Väinölä 2005).

### Microspectrophotometry (MSP)

The animals were transferred under dim red light from aquaria, where they had been kept in darkness, to a light-tight container at least 1 day before experiments to allow for proper dark-adaptation. After that, all procedures and preparations were performed under infrared light with the aid of an IR-viewer. Absorbance spectra were recorded from single rhabdoms with lateral incidence of light polarized perpendicularly to the long axis of the rhabdom. For detailed descriptions of recording and analysis, see Jokela-Määttä et al. (2005). It should be noted that our MSP does not allow us to isolate the absorbance of single photoreceptor cells (rhabdomeres), but provides the summed absorbance of several or all rhabdomeres in the rhabdom.

Spectra were basically similar in shape and could thus be characterized by a single parameter, the wavelength of maximum absorbance (*λ*_max_), obtained by fitting the data with standard templates of Govardovskii et al. (2000) (hereafter referred to as GFRKD). Following the practice of Jokela-Määttä et al. (2005) and Audzijonyte et al. (2012) we used the single-parameter A2 template as a phenomenological descriptor, which provided good fits and yielded *λ*_max_ in standardized fashion. However, as we now conclude that the broad “A2-like” spectra in fact arise from mixtures of two A1 pigments, we demonstrate that equivalent fits can be obtained with sums of two A1 templates in different proportions (Fig. 2a). While this allows spectral identification of the two component pigments, it is less useful for descriptive purposes, as fitting entails fixing three parameters: *λ*_max_ of both components, and the proportion of the two, leaving considerable room for trade-offs.

The MSP experiments involving spectrally selective bleaching of the visual pigments were carried out in the presence of hydroxylamine (pH 6.5, 50 mM) to prevent formation of metarhodopsins that would obscure the spectra of the native rhodopsins. Hydroxylamine binds the chromophore of the photoconverted pigment, forming an oxime with sufficiently short-wavelength-shifted absorbance spectrum not to interfere significantly with the peaks of the native visual-pigment spectra. For a detailed description of this method, the reader is referred to Zak et al. (2009).

### Light measurements in the habitats of the populations

Absolute irradiance spectra were measured at several depths at locations #5–11 with an OceanOptics JAZ portable spectrometer and a 20 m long optic cable with cosine collector. Measurements were done with the sensor directed both upwards and downwards, first just above the water surface and then at 1 m intervals down to a depth of 18 m. We eliminated the effect of drift on the depths of measurements either by attaching a weight to the cable or by taking the angle of the cable into account in the calculations. A mean spectrum was calculated from the “up” and “down” measurements and smoothed by Gaussian convolution. The wavelength of maximal transmission of light (WMTL) was calculated as the mean of the peak wavelengths of all measurements where spectra rose above baseline noise in a wavelength range from 423 to 747 nm. The light attenuation coefficient *k* was determined by fitting an exponential to the intensity values at WMTL as function of depth. The transmitted light at WMTL at depth *x* below surface is *I*(*x*) = *I*_0_exp(−*kx*) where *I*_0_ is the intensity at the surface and *x* is depth in meters. The wavelength of mean transmission (MT) at different depths was calculated as the wavelength bisecting the area under the transmission spectrum (i.e., so that the integrated photon fluxes are equal above and below that wavelength). These values are given in Table 1.

**Table 1:** Summary of *Mysis* single-rhabdom absorbance spectra and water transmission properties for 15 populations of glacial-relict species from Fennoscandian lakes and different parts of the Baltic Sea, and for 3 *M. mixta* populations from the Baltic Sea (see map in Fig. 1b)

	Location	Species	*λ*_max_ (nm)	WMTL	MT	*k*	*N*	*n*	Opsin lineage^b^
1	Mjörn	*M. salemaai*	556.5 ± 1.2^a^	585	611	−0.7	5^a^		II
2	Båven	*M. relicta*	555.5 ± 0.3^a^	579	582	−0.5	5^a^		II
3	Klovaskär	*M. salemaai*	525.4 ± 0.3^b^	565^c^	562	−0.4	6^b^	18^b^	I/II
4	Bothnian bay	*M. salemaai*	521.2 ± 0.4^b^	580^d^			3^b^	19^b^	I/II
		*M. relicta*	535.0 ± 1.2^b^				2^b^	21^b^	II
5	Vuohijärvi	*M. relicta*	556.0 ± 0.4^b^	584	610	−0.5	7^b^	15^b^	II
6	Pulmankijärvi	*M. segerstralei*	562.1 ± 0.3^b^	583	600	−0.6	12^b^	5	I
7	Pyhäjärvi	*M. relicta*	536.6 ± 2.6	583	616	−0.7	3	22	
8	Mahlunjärvi	*M. relicta*	563.2 ± 0.9	700	682	−1.6	9	15	
9	Kukkia	*M. relicta*	560.6 ± 0.5	648	637	−1.0	9	21	
10	Pääjärvi	*M. relicta*	561.0 ± 0.3	686	668	−1.2	3	25	
11	Pojoviken	*M. relicta*	535.0 ± 2.0	582	594	−0.8	9	17	II
12	Bothnian Bay south	*M. salemaai*	526.2 ± 0.8	555^d^			10	19	
		*M. mixta*	517.1				1	25	
13	Sea of Åland	*M. salemaai*	523.5 ± 1.3	560^d^			4	25	
		*M. mixta*	520.6 ± 0.1				2	25	
14	Gulf of Finland	*M. relicta*	550.1 ± 1.7	565^d^			2	16	
		*M. salemaai*	528.5 ± 2.9				3	16	
		*M. mixta*	524.5 ± 2.7				2	10	

Opsin lineage: different opsin genes interpreted as alleles of the same locus by Audzijonyte et al. (2012). See “Discussion”

Superscripts: data reported in ^a^Jokela-Määttä et al. (2005), ^b^ Audzijonyte et al. (2012), and ^c^ Audzijonyte et al. (2005), and ^d^ estimates based on literature

*λ*_max_ (nm), mean ± SEM of *λ*_max_ of all individuals studied in a population, *WMTL* wavelength of maximal transmission of light in the water column; MT, wavelength of mean transmission; *k* (m^*−1*^), attenuation coefficient of light in the water column. The intensity of light at WMTL as function of depth below surface is *I*(*x*) = *I*_0_ exp(−*kx*) where *x* is depth in meters and I_0_ is the intensity at the surface; *N*, number of individual animals studied; *n*, mean number of rhabdoms measured per individual

### Calculation of quantum catch and conceptual signal-to noise ratio of rhodopsins as function of *λ*_max_ in a given light environment

Relative quantum catch (QC) as function of *λ*_max_ for A1 visual pigments [QC_rel_(*λ*_max_)] was calculated for the two well-studied populations *L*_p_ (#10) at 1 and 3 m depth below surface, and *S*_p_ (#11) at 4 and 8 m depth below surface. In lake habitat #10, 3 m was the deepest point where a proper transmission spectrum could be measured; for sea habitat #11, the depths chosen were those where the absolute irradiances matched those at 1 and 3 m in #10. Relative quantum catch QC_rel_(*λ*_max_) was obtained by convolution of normalized light transmission spectra with GFRKD A1 templates. The conceptual signal-to-noise ratio of the visual pigment as function of *λ*_max_, SNR_dark_(*λ*_max_), was calculated as described in Jokela-Määttä et al. (2007). Briefly, the pigment signal is QC_rel_(*λ*_max_) in the particular light environment, and the pigment noise is the Poisson standard deviation of its rate of spontaneous thermal activations, *F*(*λ*_max_), as given by Ala-Laurila et al. (2004; Eqs. 4 and 8). The Poisson standard deviation is the square root of the mean, thus SNR_dark_(*λ*_max_) = QC_rel_(*λ*_max_)/√*F*(*λ*_max_) (when both photoactivations and thermal activations are integrated over the same time interval).

### ERG recording

Whole eye field potentials (electroretinograms, ERG) in response to flashes of light were recorded as described by Lindström and Nilsson (1988) and Pahlberg et al. (2005). Before experiments, the animals were kept in the dark and all preparation procedures were carried out under infrared light with the aid of an IR-viewer. After decapitation of the animal, the head was mounted in the specimen chamber, where it was bathed in brackish water from the Baltic Sea (salinity ~0.5 %). ERG responses were DC recorded (bandwidth 0–300 Hz) at 5 °C. Under these conditions, the preparation was very stable even for 24 h. The Inset in Fig. 6 shows the geometry, recordings being done from one of the eyes in situ on the head. An extracellular glass pipette (tip diameter ca 10 µm) filled with 100 mM NaCl and equipped with an Ag/AgCl electrode was advanced to a depth of 40–50 µm through a small hole made by a microneedle in the cornea of the dorsal region, while an Ag/AgCl wire in the bath served as reference.

The ERG was used for studying the polarization sensitivity of the eye and its correlation with short- and long-wavelength-sensitive response components. The stimulus protocol involved continuous alternation of flashes from a blue (460 nm) and a red (630 nm) LED. A linear polarizer was inserted above the eye perpendicularly to the beam, which impinged on the eye perpendicularly to its dorso-frontal surface (see inset in Fig. 6). The polarizer was rotated through 360° in steps of 9° after each pair of blue and red flashes. The intensities of the blue and red LEDs were initially set to elicit responses of equal amplitude at the “start” (0°) orientation of the polarizer.

Since estimation of photoisomerization rates in the intact *Mysis* eye is difficult due to screening pigments and eye geometry, and since absolute values were not needed here, flash intensities (*I*_F_) are expressed relative to a “threshold” flash intensity (*I*_FT_), defined as that which (in each preparation) elicited an ERG criterion response of 20 μV in the dark-adapted state. Both LEDs were driven at constant power, and flash intensity (strictly speaking, photon dose) was varied using pulses of four different durations: 0.02, 0.2, 2.0, and 20 ms. The longest pulse is not “an infinitely brief flash” from the viewpoint of response shape, but this entailed no disadvantage, as minor changes in response shape with changes in the length of brief pulses are of no concern here. Given the limitations of our light sources, this was an acceptable way of getting up high enough on the (saturating) response-amplitude vs. stimulus-intensity function.

### Determination of chromophore identity

Chromophore identity was determined by HPLC as described in detail in Belikov et al. (2014). Briefly, eye extracts were run against references consisting of the same extracts to which had been added either pure A1 or pure A2 chromophore. The added chromophores produced significant and easily identifiable peaks (Fig. 4).

### Ethical statement

None of the mysid species studied is endangered in the sampling areas (see map in Fig. 1b). *M. relicta* (*sensu lato*) are in fact the most common macrocrustaceans in Finnish waters. Under Finnish legislation, no permit is needed for sampling of invertebrates. Public access to all areas is guaranteed according to the general principle of “everyman’s right” (common rights) regardless of ownership (private/state/municipal), unless explicit and precisely specified regulations apply (which is not the case here). Common rights include unrestricted sampling of such invertebrate species that are not defined as endangered, and the land owner’s permission is not required for these purposes.

## Results

### Bimodal distribution of single-rhabdom absorbance spectra in the glacial-relict mysids

Absorbance spectra were recorded by MSP from single dark-adapted rhabdoms of animals from 15 populations representing all three European glacial-relict *Mysis* species (see Fig. 1b). Figure 2a shows examples of spectra from single individuals of five populations of *M. relicta*. Each row represents one population/habitat. Rows 1–4 from top roughly span the optical range of fresh-water habitats encountered, from “dark brown” to “clear greenish” as initially classified by visual inspection. Row 5 represents the coastal Baltic Sea. The first two columns in each row show the same spectra (averaged from 10 to 20 single-rhabdom recordings in each case) fitted with different curves. The blue and red curves in column 1 are GFRKD templates for, respectively, pure A2 and pure A1 pigments The blue curves (A2 templates), used previously by Jokela-Määttä et al. (2005) and Audzijonyte et al. (2012), are seen to provide good fits. By contrast, the red curves (A1 templates), here locked to the same *λ*_max_, are systematically too narrow. This would in no way improve if the fits were based instead on the long-wavelength limb of the spectrum, as often preferred for spectra of lower quality.

**Fig. 3:**
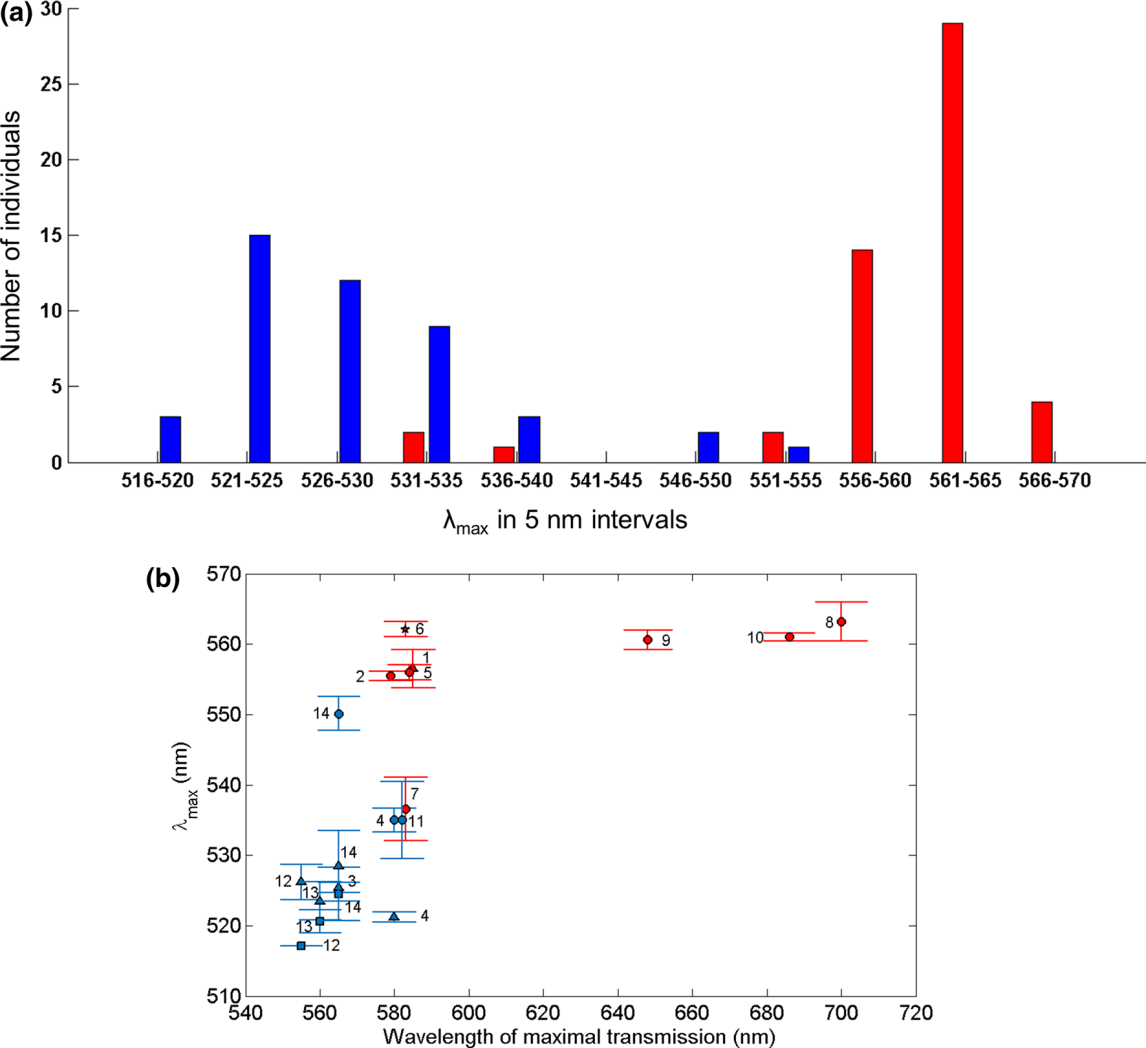
**a** Distribution of *λ*_max_ values of individual animals from six lake (*red*) and six sea (*blue*) populations (locations/populations #3–14 in Fig. 1b; Table 1). These mainly constitute two separate clusters. The exceptions (*red bars* in the short-wave cluster and *blue bars* in the long-wave cluster) are *M. relicta* individuals from location #7 (Lake Pyhäjärvi, three individuals) and locations #11 and #14 (Baltic Sea at the south coast of Finland, 1 + 2 individuals), respectively. **b** Mean *λ*_max_ ± SD for all the 19 *Mysis* populations listed in Table 1, plotted as function of the wavelength of maximum transmission of light (WMTL) in the respective habitats. The values have been calculated from all individual mean values within each population. The numbers correspond to the map in Fig. 1b and Table 1; in cases where there are several species from the same location, the species are distinguished by *different symbols*: *circles* *M. relicta*, *triangles* *M. salemaai*, *squares* *M. mixta*. While there is no clear correlation between *λ*_max_ and WMTL, all populations in brown lakes (*rightmost points*) have L-type spectral sensitivities

Column 2, however, shows that good fits can also be achieved on the assumption that spectra represent the summed absorbance of two A1 pigments (*P*_I_ and *P*_II_). Below, we argue that this is the true situation. Fitting sums of templates is less attractive if the purpose is only to provide a simple empirical description, however, since it involves three parameters: *λ*_max_(*P*_I_), *λ*_max_(*P*_II_) and the ratio (*P*_I_:*P*_II_) (see the legend to Fig. 2a), with significant room for trade-offs. Therefore, the single-parameter GFRKD A2 template was used throughout as a phenomenological descriptor for the purpose of determining *λ*_max_ of recorded spectra in a standardized way, commensurate also with the values given by Jokela-Määttä et al. (2005) and Audzijonyte et al. (2012) (Fig. 3; Table 1).

Column 3 shows the light spectrum (black) measured in each habitat at 1 m depth together with the single-rhabdom absorbance spectrum (light gray) of the corresponding sample. The vertical line drawn at 545 nm in all panels makes it easy to see, e.g., that the spectral absorbance of the population in row 1 (Lake Pääjärvi, *L*_p_) is significantly red-shifted compared with that of the population in row 5 (Pojoviken of the Baltic Sea, *S*_p_), and that the difference seems to correlate with the difference between the spectral light transmission of the two habitats. Indeed, rows 1 and 5 represent the two populations *L*_p_ and *S*_p_ where an “adaptive” shift in eye (ERG) spectral sensitivity was originally described by Lindström and Nilsson (1988).

The situation becomes much less clear, however, when the three other lakes in Fig. 2a (rows 2–4) are considered. Their spectral transmissions differ rather little from that of Pojoviken, yet the absorbance spectra recorded from animals from two of them (including the least red-shifted Lake Vuohijärvi, row 3 from top) are similar to *L*_p_, and only the Lake Pyhäjärvi population (row 4 from top) resembles *S*_p_. In fact, the last-mentioned is the only one among all eight fresh-water populations studied here that did not have the long-wavelength-shifted (*L*_p_) type of absorbance spectrum.

Figure 2b shows the general light attenuation as function of depth in the habitats included in Fig. 2a and several others from Table 1. The lakes labeled “clear/greenish” in panel (a) are similar to the Baltic Sea location Pojoviken not only in the spectral distribution of light, but also in the relatively high general light transmission. Note particularly that Lake Vuohijärvi is not only spectrally similar to Pojoviken, but has even clearer water. Only the Baltic Sea location #3 is clearer.

The consistent lake/sea (henceforth *L*/*S*) dichotomy in absorbance spectra, which overrides most specific correlations with illumination spectra, is illustrated by Fig. 3. Panel (a) shows the distribution of individual *λ*_max_ values across six *L* populations (red) and six *S* populations (blue) representing all three species, panel (b) shows population means ± standard deviations of all 19 populations in Table 1 (i.e., including also *M. mixta* besides the three glacial-relict species) as functions of the WMTL in the habitat.

The *λ*_max_ values form two distinct clusters centered on ca 530 and 560 nm, respectively (Fig. 3a). Six *M. relicta* individuals (three “red” from location #7 and three “blue” from locations #11 and #14 in Fig. 1b) fall in the “wrong” cluster, but even so there remains an empty *λ*_max_ interval without a single individual (540–545 nm). The bimodality appears even crisper in terms of population means, summarized in Table 1 (mean ± SEM) and Fig. 3b (mean ± SD). There is no population with mean *λ*_max_ in the interval 537–550 nm, but two *M. relicta* populations, #7(*L*) and #14(*S*), fall in the “wrong” group. Moreover, if the generic association of S-type sensitivity with brackish-water environments and L-type sensitivity with fresh-water environments is factored out, there remains at most a hint of residual correlation of population mean *λ*_max_ with WMTL (Fig. 3b). To appreciate the dominance of the general *L*/*S* dichotomy, it may be noted that *within* either the *L* or the *S* population group, the maximal difference in mean *λ*_max_ between two different species, *M. relicta* and *M. salemaai*, is only 14 nm in spite of substantial divergence of their opsin genes (Audzijonyte et al. 2012). By contrast, the *L*_p_ and *S*_p_ populations of *M. relicta* differ by 26 nm, although no opsin gene differences translating into amino acid substitutions have been found.

Having established the nearly complete *L*/*S* dichotomy in 15 populations of three species (Fig. 3; Table 1), we proceeded to identify molecular and cellular mechanisms in one well-established model population pair (*L*_p_ and *S*_p_) of one species, *M. relicta*. We shall argue that the conclusions are likely to hold for all populations across species.

### The bimodal distribution of single-rhabdom absorbance spectra is not due to a chromophore difference

The most obvious hypothesis for explaining a pervasive 20–30 nm spectral shift between *L* and *S* populations would be that they use different chromophores in the same opsin. The A1 ↔ A2 (rhodopsin–porphyropsin) system underlies “fast” tuning of spectral sensitivity in fishes and amphibians (e.g., Schwanzara 1967; Bridges 1972; Enright et al. 2015), and is known to be used also by at least one crustacean species, the fresh-water crayfish *Procambarus clarkii* (Suzuki et al. 1984, 1985, 1993). In *Procambarus*, like in a great number of vertebrate species, the balance of the two chromophores is regulated by environmental factors, primarily light and temperature (Suzuki et al. 1985). In the *λ*_max_ range relevant here (520–560 nm), replacing A1 by A2 in the same opsin is expected to cause a red shift of about the right magnitude (~30 nm) (Dartnall and Lythgoe 1965; Hárosi 1994).

**Fig. 4:**
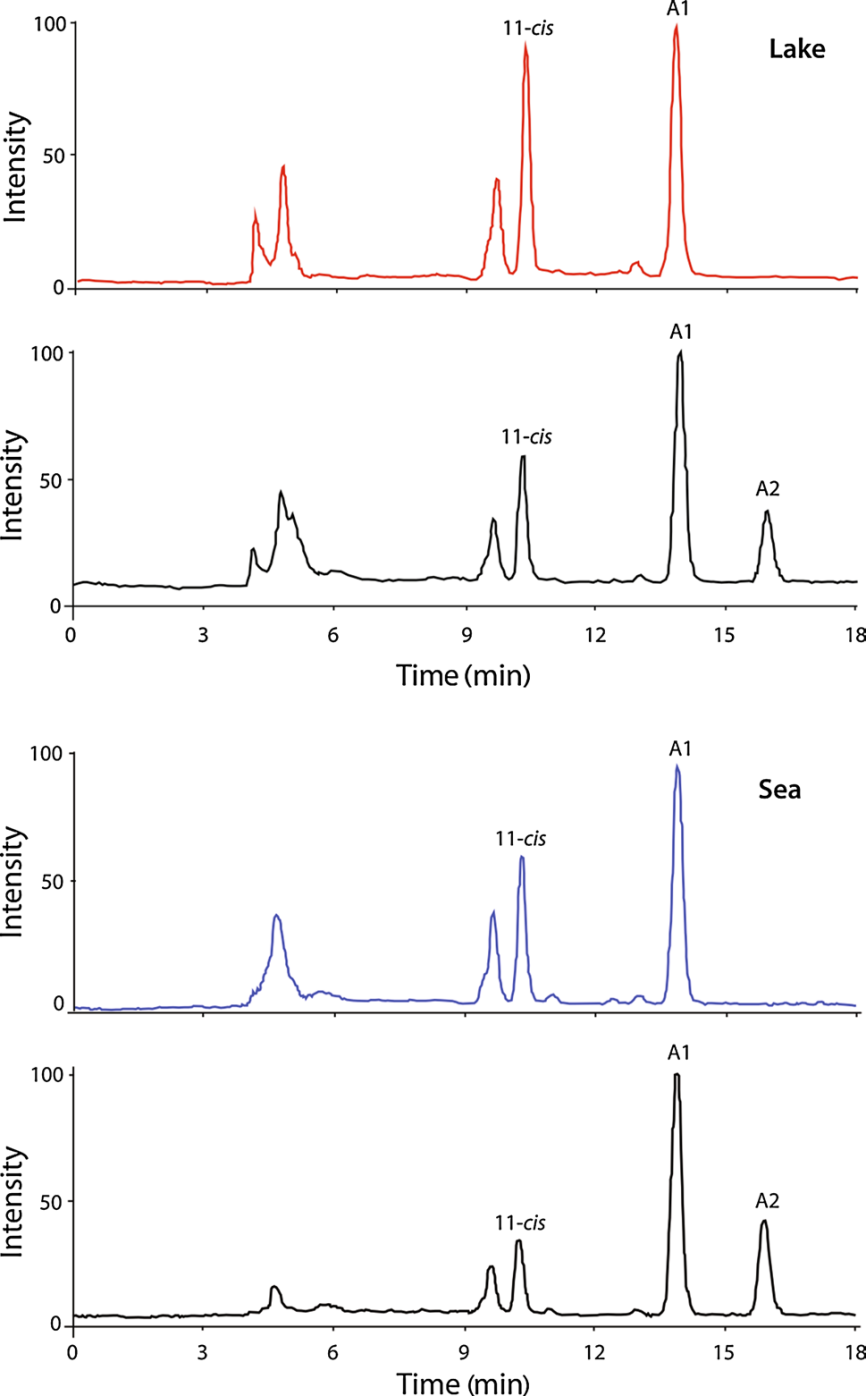
HPLC analysis of eye extracts of *L*_p_ (*red trace*, *top*) and *S*_p_ (*blue trace*, *bottom*) *M. relicta*. Each extract is compared with the same extract to which have been added pure chromophores A1 and A2 (*black traces*). In neither population does the native eye extract show any signal corresponding to the A2 peak in the black reference trace (Belikov et al. 2014); hence, both use only chromophore A1

Chromophore identity was determined by HPLC in our main model population pair *L*_p_ and *S*_p_ (#10 and #11 in Table 1; Fig. 1b) as described by Belikov et al. (2014) (see “Materials and methods”). Eye extracts were run against the same extracts supplemented with pure A1 and A2 chromophores. The unambiguous result (Fig. 4) is that the eyes of both populations contain only A1, with no measurable trace of A2. There are good reasons to think that this conclusion can be generalized to all populations and species (see “Discussion”).

### The different absorbance spectra of *L*_p_ and *S*_p_*M. relicta* are due to different proportions of two rhodopsins

As shown in Fig. 2, the “A2-like” broad shape of the absorbance spectra can be generated by sums of A1 templates. Since the eyes contain only A1 chromophore, the spectra must indeed arise from mixtures of A1 pigments. In order to investigate directly whether two rhodopsins differing in *λ*_max_ are present we performed experiments where changes in MSP absorbance spectra were measured at different stages of spectrally selective bleaching of single rhabdoms. These experiments were done in the presence of hydroxylamine, which will remove metarhodopsins (dominant *λ*_max_ ~490 nm) liable to obscure the absorbance spectra of remaining native rhodopsins. Under these conditions the final bleaching product is opsin and retinal oxime, which absorbs at wavelengths short enough not to interfere significantly (*λ*_max_ < 390 nm; see Bridges 1972; Zak et al. 2009; cf. “Materials and methods”).

**Fig. 5:**
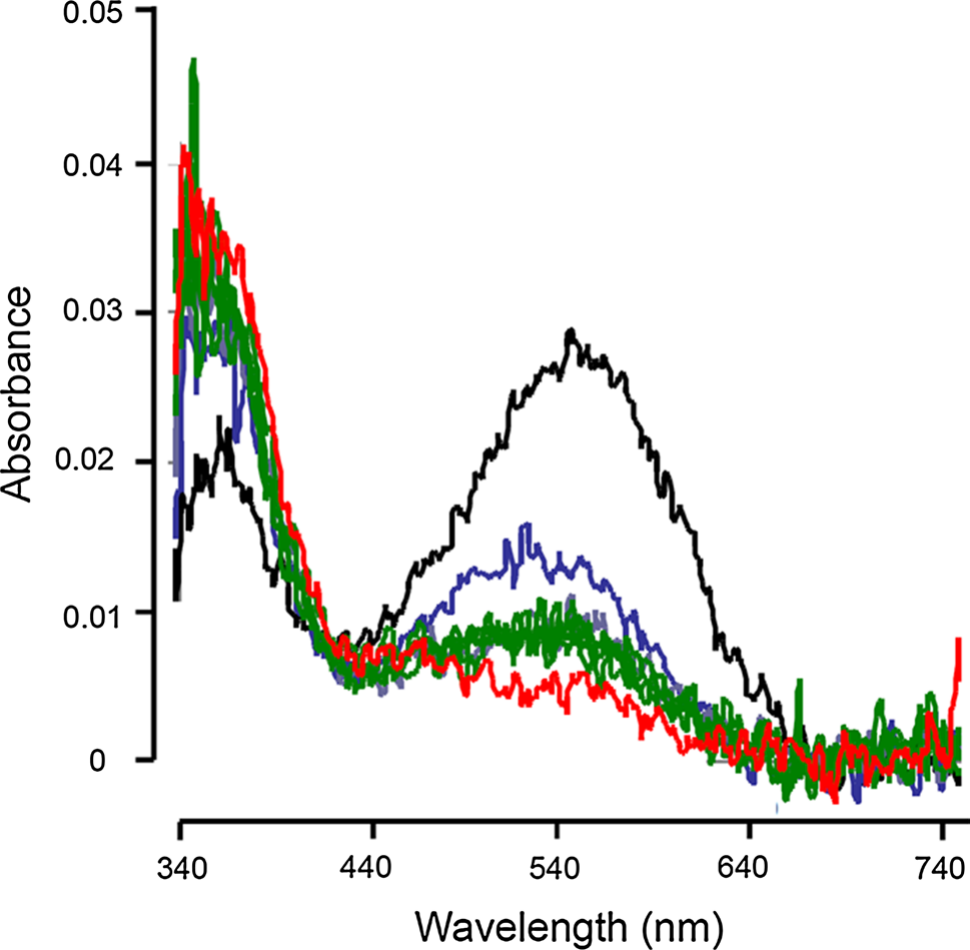
MSP absorbance spectra recorded from a single *L*_p_ rhabdom at different stages of extended exposures to strong 650 nm (several epochs) and thereafter 560 nm light in the presence of hydroxylamine, which results in spectrally selective bleaching of the visual pigments. *Black curve* dark adapted state at the beginning of the experiment; *blue curve* after bleaching with 650 nm light for 340 s; *green curves* semi-steady state after bleaching with 650 nm light for totally 1140 s (340 + 300 + 150 + 100 + 150 + 100 s), *red curve* final state after additional bleaching with 560 nm light for 300 s (100 + 100 + 100 s). The hydroxylamine serves to remove metarhodopsins, the absorbance spectra of which would overlap with those of native rhodopsins. The rise in absorbance below 400 nm is due to the retinal oxime formed as hydroxylamine binds the A1 chromophore. (*λ*_max_ ≈ 368 nm; see Bridges (1972))

Figure 5 shows a family of absorbance spectra of an *L*_p_ rhabdom recorded by MSP at different stages of partial bleaching first with long-wavelength light (650 nm), then with shorter wavelength light (560 nm). Several epochs of 650 nm bleaching not only depressed the overall absorbance in a graded manner, more importantly, it shifted the absorbance peak towards shorter wavelengths (blue and green traces). After 640 s of bleaching, a low peak (green) remained around 530 nm that was virtually unaffected by 500 s of further exposure to 650 nm light. However, a final phase of 560 nm exposure made the peak disappear completely (red trace), confirming its origin in a second, bleachable visual pigment. The *λ*_max_ and amplitudes of the two bleachable spectral components were read at the points where the distance between the spectra prior to and after extended bleaching is greatest. For the main component, this is the point of greatest distance between the black curve and the curve unresponsive to extended 650 nm exposure (taken as the middle of the many superimposed recordings seen as a broad green noise band), at ca. 565–570 nm. For the second component, this is the point of greatest distance between the latter and the final photostable baseline (red), at ca. 530–535 nm. The amplitude ratio is roughly 5:1. This might in principle be translated into a molecular ratio of a long-wavelength sensitive (LWS) and a middle-wavelength-sensitive (MWS) rhodopsin with *λ*_max_ ≈ 565–570 and 530–535 nm, respectively. The best fits for combinations of two A1 pigments, based on GFRKD templates, across all *M. relicta* populations, when the two components are constrained to have the same *λ*_max_ values in all (cf. the fits in the second column of Fig. 2), suggested that LWS and MWS *λ*_max_ might lie even further apart (570 and 525 nm). These estimates are fraught with considerable uncertainty, however. Given that no differences in the opsin genes translating into amino acid differences have been detected between *L*_p_ and *S*_p_ (Audzijonyte et al. 2012; see “Discussion”), we attribute the variability to technical limitations.

Experimental isolation of MWS and LWS components by spectrally selective bleaching could not be successfully performed on *S*_p_ rhabdoms. Spectral changes even due to full bleaching of the minority (LWS) pigment are difficult to measure reliably, and the majority (MWS) pigment cannot be selectively targeted without simultaneous bleaching of the LWS pigment. Figure 5 therefore, essentially offers a “proof of principle”, i.e., experimental support for the idea that the broad A2-like spectra can be decomposed into two A1 spectra. For a comprehensive picture of pigment composition in all populations (*L* as well as *S*) of all species we relied on A1/A1 template-fitting to unbleached rhabdoms. A cautious conclusion is that the two components of *M. salemaai* lie at ca. 10 nm shorter wavelengths compared with those of *M. relicta*, best estimates being *λ*_max_(*P*_LWS_) ≈ 560 and *λ*_max_(*P*_MWS_) ≈ 515. The estimated (*P*_LWS_:*P*_MWS_) ratios are even more uncertain, as they depend strongly on the choice of *λ*_max_, but for the given *λ*_max_ values, the fitting suggests ca. 3:1 in *L* populations and 1:3 in *S* populations.

### The two rhodopsins are segregated into different cells with different polarization sensitivities

Given that *M. relicta* possesses two spectrally different visual pigments, their function will crucially depend on whether they are homogeneously mixed in the same photoreceptor cells (albeit in different proportions in different populations), or partly or wholly segregated into different cells. In the former case, adjusting their proportions would only serve tuning of the overall spectral sensitivity of the eye; in the latter case they could also form the basis for dichromatic wavelength discrimination. Our MSP technique does not generally allow us to resolve the absorbance of single photoreceptor cells within a rhabdom. Therefore, we studied this question electrophysiologically by ERG, using polarized light for stimulation.

A common paradigm for separation of visual mechanisms with different spectral sensitivities is using spectrally selective light-adaptation (Stiles 1949). This route was taken by Zak et al. (2013), who found that “red” adaptation desensitized “red” responses more strongly than “blue” responses and took this as evidence that pigment proportions differ between cells. If all cells contained the same mixture, they argued, response changes would be independent of the wavelengths of adapting and testing lights and depend only on the rates and numbers of photoisomerizations from the background and the probe flash (the principle of univariance). While this conclusion would be valid for vertebrates, the evidence may not be compelling for arthropods, where bidirectional photoconversion between rhodopsin and (shorter wavelength sensitive) metarhodopsin is common. “Red” adaptation might then suppress “red” responses relative to “blue” responses just because the rhodopsin:metarhodopsin ratio changes.

Here we approached the question by studying wavelength-dependent differences in the polarization sensitivity of the eye. Arthropod photoreceptors potentially support discrimination of polarization angles, as light with the e-vector parallel to the microvilli is best absorbed. Photoreceptor cells with microvilli well-oriented in one plane will exhibit strong polarization selectivity, whereas photoreceptor cells with microvilli oriented in several directions, or higher order neurons summing responses from several photoreceptors with different microvillar orientations, will show little or no polarization selectivity. Thus comparison between (groups of) photoreceptors differing in microvillar orientation may form the basis for polarization discrimination (see, e.g., Fein and Szuts 1982).

We tested for polarization selectivity in general, and specifically for possible differences in the polarization preference of LWS- vs. MWS-driven responses, by presenting the eye with alternating flashes of linearly polarized “blue” (460 nm) and “red” (660 nm) light. The blue and red flash intensities were initially set to elicit responses of equal size at one orientation of the linear polarizer (relative log flash intensities *I*_F_ set such that log *I*_F_/*I*_TF_ = 3, see “Materials and methods”). Blue and red flashes of these fixed intensities were alternated at 0.3 s intervals, while the polarizer was turned through 360° in 9° steps after each blue-red flash pair.

**Fig. 6:**
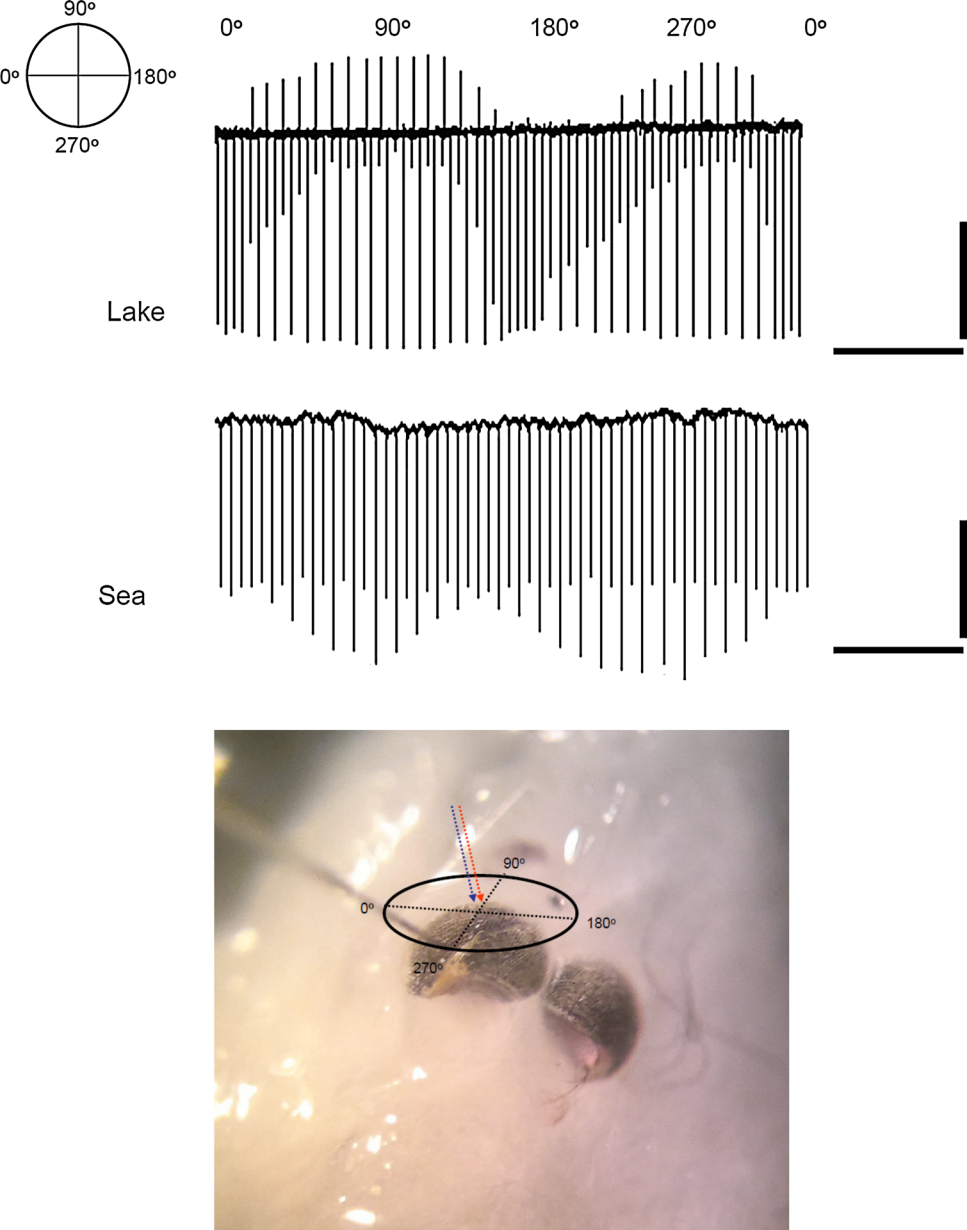
Changes in ERG responses of *L*_p_ and *S*_p_ eyes to alternating red and blue flashes of constant intensity as function of the plane of linear polarization of the stimulating light. *Inset* recording configuration. *Top panel* responses of *L*_p_ eye. The angular orientation of the polarizer is indicated above the trace. *Red* and *blue flashes* alternate; the responses that change as function of the plane of polarization are responses to *blue flashes*. The amplitudes of responses to *red flashes* are approximately constant. *Scale bars* 400 μV (*vertical*) and 5 s (*horizontal*). *Bottom panel* responses of *S*_p_ eye. The responses that change as function of the plane of polarization are responses to *red flashes*. The amplitudes of responses to *blue flashes* are approximately constant. *Scale bars* 100 μV and 5 s. Initially the *red* and *blue flashes* were set to give responses of equal amplitude at one orientation of the polarizer (marked 0° or 180°), as seen for the first two and final two flash pairs in both records. Flash intensities were 3 log units above the respective threshold intensities (log *I*_F_/*I*_TF_ = 3; the absolute intensity values were different for *red* and *blue* as well as for *L*_p_ and *S*_p_). See Text for further details

Recordings from both an *L*_p_ and an *S*_p_ eye are shown in Fig. 6. The eyes of both populations showed clear polarization sensitivity correlating with flash wavelength. In *L*_p_, the blue response was strongly polarization-dependent (Fig. 6, top trace). It changed in both amplitude and shape, indicating changes not only in the strengths of underlying currents, but also a geometrical redistribution. This might suggest more complex interactions between cell types, but given the approximate constancy of the red response, the most parsimonious interpretation is that the polarization sensitivity originates in the MWS cells. In *S*_p_, on the contrary, it was the red response, mediated by LWS cells, that showed polarization-dependence (Fig. 6, bottom trace), whereas the blue response (mainly from MWS cells) stayed approximately constant.

Summarizing, it appears that the cells dominated by the minority pigment are the ones that primarily mediate polarization discrimination in both populations (MWS in *L*_p_ and LWS in *S*_p_), presumably by having microvilli well-oriented in a single plane. It may be noted that the best differentiation in both cases occurred for light polarized in the sagittal plane of the animal. With respect to precise interpretation of the ERG results, we need to make two cautionary remarks, however. First, even in case red responses originate exclusively in LWS cells, responses to blue cannot originate exclusively in MWS cells, but will necessarily contain an LWS-cell component because of the overlap of spectral sensitivities. Second, the ERG is an ohmic field potential reflecting changes in all appropriately oriented extracellular currents as well as resistance changes in the eye tissue, and the relation between cell processes and ERG voltage may be quite complex (see Donner et al. 1992).

## Discussion

### *Mysis* spectra form two clusters that mainly correlate with fresh- vs. brackish-water habitats

Our comparison of whole rhabdom absorbance spectra from a large and diverse sample of glacial-relict *Mysis* (15 populations representing three species) yielded three principal results. First, the spectra show a strongly bimodal distribution, with two non-overlapping *λ*_max_ clusters centered on ca 530 and 560 nm. Second, the shorter wavelength cluster mainly comprises brackish-water populations (“sea”, *S*) and the longer wavelength cluster fresh-water populations (“lake”, *L*), with only two exceptions (one *L* and one *S* population). Third, within the clusters there is no consistent correlation of mean population *λ*_max_ and the spectral properties of the habitat. Against this general background, we have endeavored to dissect underlying mechanisms in one *S*/*L* population pair, *M. relicta* from Pojoviken of the Baltic Sea and from Lake Pääjärvi (populations *S*_p_ and *L*_p_; #11 and #10). We argue that the mechanistic conclusions from this model pair can be generalized to all species and populations, and this leads to a new hypothesis regarding the origin of the bimodal *λ*_max_ distribution.

### Two A1 pigments are expressed in different proportions not correlating with the light environments

First, it was shown that the spectral difference between *L*_p_ and *S*_p_ rhabdoms is not due to different chromophore usage, but that both use only the A1 chromophore (Fig. 4). We think that this result is likely to hold for all species/populations considered here. They have all experienced similar challenges involving repeated switches between fresh- and salt water conditions and would presumably have had continuous use for the chromophore exchange mechanism, had it been available. Had there been an ancestral A1 ↔ A2 system (which would have been retained in some of the species/populations), it would hardly have been lost specifically in the particular line leading to our model *M. relicta* population pair, which would then (in a remarkably short time) have reinvented a similar spectral shift by a different mechanism. An almost equally unlikely scenario is that the special biochemical machinery needed, involving a physiologically regulated dehydrogenase such as Cyp27c1 in vertebrates (Enright et al. 2015), should have evolved ab initio in some of the sibling species. Although there might in principle exist, e.g., seasonal variation that has escaped our notice (cf. for example Temple et al. 2006), it should be remembered that the only well-established example of the A1 ↔ A2 system in crustaceans is the freshwater decapod *Procambarus clarkii* (Suzuki et al. 1984, 1985, 1993), which differs from the primarily marine mysids in many respects. Thus the present result from the *L*_p_ to *S*_p_ population pair of *M. relicta* strongly suggests that none of the species or populations have the A1 ↔ A2 system for shifting spectral sensitivity.

Second, it was shown that at least the *L*_p_ population achieves the spectral difference by expressing two pigments (LWS and MWS) in unequal proportions. If it is accepted that none of the species use A2 chromophore, the similar broad shape of all spectra suggest that these can be similarly decomposed into two A1 spectra. Fitting sums of two GFRKD A1 templates to the recorded spectra of all populations, assuming that the two components are the same within the same species, yielded best estimates *λ*_max_(*P*_LWS_) ≈ 570 nm and *λ*_max_(P_MWS_) ≈ 525 nm for *M. relicta*. Several opsin sequence differences have been found between the three species *M. relicta*, *M. salemaai* and *M. segerstralei* (Audzijonyte et al. 2012). Consistent with this, the best fits of two A1 templates in *M. salemaai* were achieved with *λ*_max_(*P*_LWS_) ≈ 560 nm and *λ*_max_(*P*_MWS_) ≈ 515 nm, whilst *M. segerstralei* was close to *M. relicta*. It is worth emphasizing again, however, how emphatically the dichotomy of the *λ*_max_ distribution between the *L* and *S* groups overrides the inter-species variation of single-rhabdom absorbance spectra within the *L* and *S* groups.

### Hypothesis: a developmental reaction norm conserved from a common ancestor

The summed evidence is consistent with the hypothesis that the expression of LWS and MWS opsins is subject to phenotypic plasticity controlled by some environmental factor(s) other than light that generally differ(s) between the brackish- and fresh-water habitats. This could be anything from salinity per se to, e.g., concentrations of specific ions (Ca^2+^, Cl^−^), humic acids, or pH. Light may play some role, but is clearly not the decisive factor. Resolving these questions requires further study.

The functionality of pigments with different *λ*_max_ for dim-light vision in a particular spectral environment can be measured either by quantum catch (QC) or by the conceptual signal-to-noise ratio (SNR_dark_) of the pigment (Jokela-Määttä et al. 2007; Saarinen et al. 2012; see “Materials and methods”). QC is decisive at (somewhat) higher light levels, where the random arrival of photons (“quantal fluctuations”) is a more powerful noise source than the spontaneous thermal activations of pigment molecules. At the very lowest light levels (near the absolute visual sensitivity limit), the decisive factor is SNR_dark_, calculated as QC/√(thermal activations), where both QC and thermal activations are numbers of pigment activations per integration time (see “Materials and methods”). When plotted for a given photic environment as functions of pigment *λ*_max_, SNR_dark_ will peak at lower *λ*_max_ values than QC, because the thermal activation rates of visual pigments increase with increasing *λ*_max_ (Barlow 1957; Ala-Laurila et al. 2004; Luo et al. 2011).

**Fig. 7:**
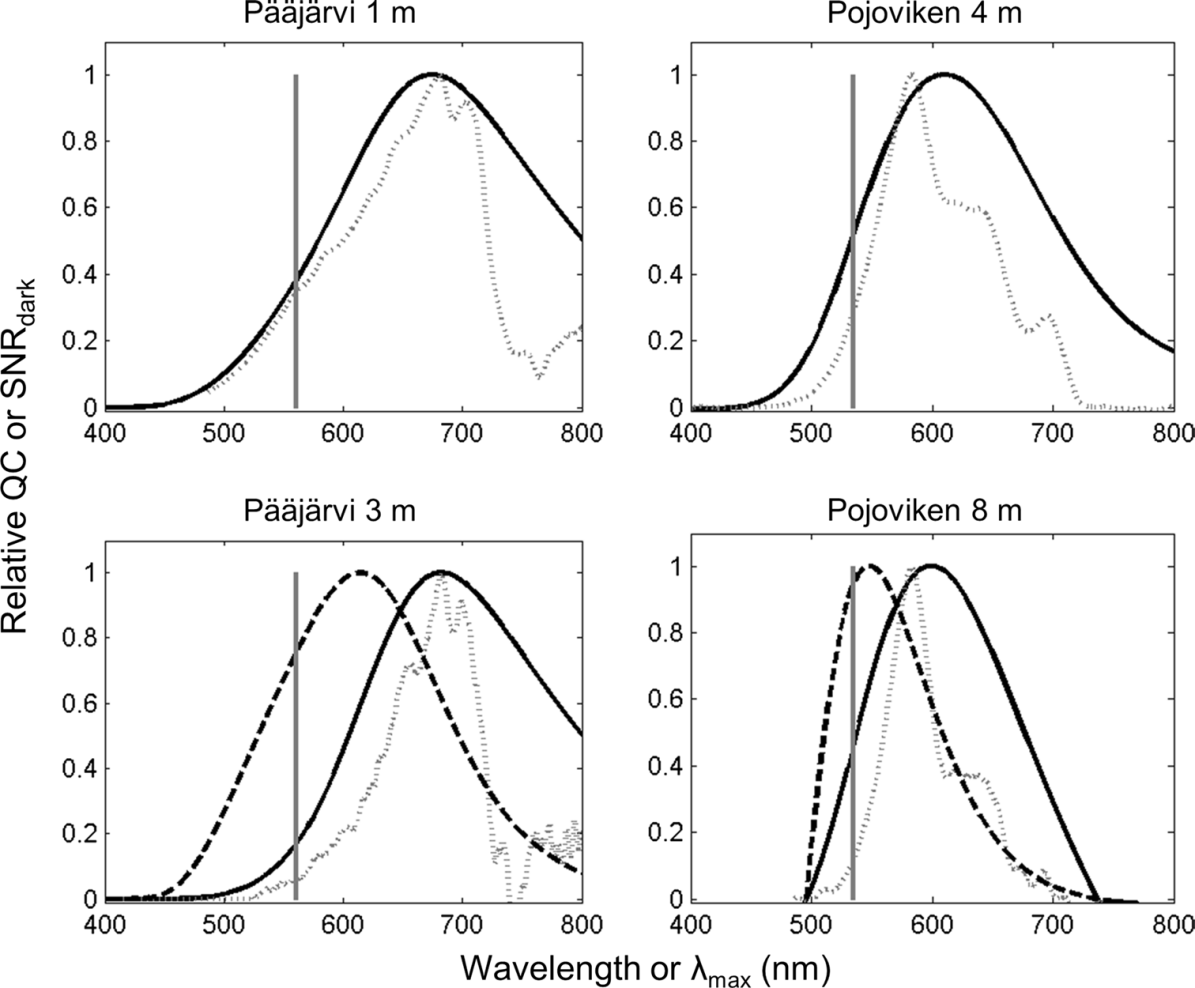
Theoretical performance of rhodopsins as function of their *λ*_max_ at two depths in the habitats of the main model population pair *L*_p_ (Lake Pääjärvi, #10 in Fig. 1; Table 1) and *S*_p_ (Pojoviken Bay, #11). The *thin dotted lines* show the recorded illumination spectra (photons m^−2^ s^−1^ nm^−1^); for these the abscissa is light wavelength *λ* (nm). The *full-drawn curves* show relative quantum catch (photon absorptions s^−1^) as function of the pigment’s *λ*_max_. The *dashed lines* in the *bottom panels* show the conceptual signal-to-noise ratio (SNR_dark_) of pigments as functions of *λ*_max_. All *curves* have been normalized to unity. The *gray vertical bars* mark the mean *λ*_max_ values actually recorded in the respective populations (Table 1)

Figure 7 shows QC (bold full-drawn curves in all panels) and SNR_dark_ (bold dashed curves in bottom panels) as functions of pigment *λ*_max_ calculated for two depths in the habitats of our two main model populations *L*_p_ and *S*_p_. The actual light spectra are shown as thin dotted curves. The first observation is that both L- and S-type *λ*_max_ (here, 561 and 535 nm, marked by vertical gray lines) fall far below that which would maximize QC in both light environments (here ca. 680 and 600 nm, respectively). This changes little with depth. In the deeper layers, however, SNR_dark_ may become more relevant than QC as such, especially at twilight or night. The SNR_dark_ curves in the bottom panels show that the optimal *λ*_max_ is significantly shifted towards shorter wavelengths. Measured by SNR_dark_ deep in the Pojoviken environment, the spectral sensitivity of *S*_p_ is nearly optimal (only slightly on the “green” side of the optimum), but neither is the *λ*_max_ of *L*_p_ far from the Pojoviken optimum (only slightly on the “red” side). From the viewpoint of conceptual signal-to-noise ratio (absolute visual sensitivity), both S-type and L-type spectra would work quite well in the Baltic Sea, whereas the performance of S-type pigments would be considerably less good in Lake Pääjärvi.

Thus a possible evolutionary scenario might be as follows. The mysids that colonized coastal and continental waters during the Pleistocene experienced repeated and partly correlated changes in chemical conditions and water color when lakes and brackish inland seas were alternately sequestered and rejoined, even to the ocean, as the land sank and rose. The mysids, a primarily marine clade, have never had the A1 ↔ A2 system for spectral tuning. Instead, they recruited a pre-existing dichromatic system, common in crustaceans (Wald 1968), for a similar purpose. The simple rule that “marine or brackish-water” conditions enhance expression of the shorter wavelength pigment and “fresh-water” conditions expression of the longer wavelength pigment may then have been, on average, a useful predictive adaptation to photic conditions, in spite of random but less frequent deviations in specific cases. Going by default for long-wavelength sensitivity in fresh water will give a great advantage if the lake is brown, but no great disadvantage if the lake happens to have Baltic-like transmission. On the other hand, in the Baltic Sea truly “brownified” environments are never encountered and shorter wavelength sensitivity works better (Fig. 7). This predictive pattern would have been genetically fixed as a reaction norm (Woltereck 1909, 1928), against which there would seem to be no significant selection pressure. It mimicks the A1 ↔ A2 system without incurring the excessive noisiness of A2 pigments (Donner et al. 1990; Ala-Laurila et al. 2007), which in terms of SNR_dark_ will easily offset or even reverse gains accruing from higher QC (cf. Saarinen et al. 2012). Most mysids live at least certain phases of their lives in very dim light, and need high absolute visual sensitivity (high SNR_dark_).

We should like to add two comments. First, the ERG experiments with polarized light (Fig. 6) indicated that LWS and MWS pigments are largely or wholly segregated into different cells with different properties. This suggests that the developmental switch controls not only the relative expression levels of the two pigments, but alternative pathways in photoreceptor development, involving both the pigments and the morphology and architecture of retinula cells. Second, the notion of an ancestral reaction norm generally conserved across species and populations does not exclude the possibility that some populations may have been fixed in either the L or the S state due to loss of function, e.g., in connection with population bottlenecks.

### Dichromatic vision and polarization sensitivity

Differences in light polarization constitute a potentially useful source of visual information even in deep aquatic environments (Cronin and Shashar 2001; Cronin 2006). Polarization sensitivity may be used for orientation, both vertical and horizontal, for detecting food (breaking transparency camouflage) as well as for signaling (Shashar et al. 2004). The degrees and patterns of polarization are fairly constant over the wavelength range 400–580 nm down to considerable depths in the water (≥15 m: Cronin and Shashar 2001). Mysids are known to use their vision at least for feeding and for assessment of depth in the water column, e.g., in connection with diel vertical migrations (Boscarino et al. 2009). Both tasks may benefit from polarization sensitivity.

The polarization-selectivity of *M. relicta* appeared to be mediated primarily by the minority-pigment cells in the respective population, i.e., MWS cells in *L*_p_ and LWS cells in *S*_p_ (Fig. 6). It is *prima facie* paradoxical that the potential for discrimination of wavelength and polarization should be coupled in the same cells. Obviously, the animal cannot then know whether a perceived contrast is due to a difference in color or polarization. Functionally, this suggests that disambiguation of wavelength and polarization contrasts has not been important, but that both serve the same tasks, be it prey detection or depth judgment (cf. Cronin 2006). Mechanistically, it supports the notion that pigment expression and cell morphology are under the control of the same developmental switch.

### Why have previous studies concluded that species of the *Mysis relicta* group possess only one opsin?

The experiments with spectrally selective bleaching provide direct evidence for the presence of two visual pigments in the rhabdoms. Thus it is puzzling why DNA studies have failed to pick up more than one opsin gene in these populations. We wish to suggest the possibility that the genetic data may have been misinterpreted. Audzijonyte et al. (2012) did find two deeply diverged opsin lineages (“haplotypes” or “alleles”) I and II, in their set of 35 populations representing all four glacial-relict species (cf. Table 1). They give several reasons for thinking that these are alleles of the same locus rather than, e.g., results of gene duplication, but none of them seems to be ultimately compelling. We tentatively suggest that the two “haplotypes” may in fact represent two opsin genes present and expressed in all the populations included here, although for unknown reasons differentially and seemingly randomly amplified in the DNA studies. In the study of Audzijonyte et al. (2012) most populations seemed to possess just one of these, but the genetic I/II dichotomy does not correlate with the phenotypic *S*/*L* dichotomy (see Table 1). In only two of the populations included here (*M. salemaai* from “sea” localities #3 and #4 in Fig. 1b) both haplotypes were found, but interestingly, these populations have pure S-type absorbance spectra and low inter-individual variability (population mean ± SEM *λ*_max_ = 521.2 ± 0.4 and 525.4 ± 0.3 nm), with no suggestion of either intermediate *λ*_max_ values or increased polymorphism.

We further note that the deep divergence of haplotypes I and II, estimated to have occurred before the speciation events, would be consistent with our hypothesis of two pigments present in all species but differentially expressed according to a conserved reaction norm. The idea of two opsin genes could also resolve some of the paradoxes encountered in the comparison of mtDNA phylogeny with opsin gene phylogeny under the single-opsin-gene assumption (Audzijonyte et al. 2012).

### *Mysis diluviana*

The fourth of the glacial-relict *Mysis* sibling species, the North American *M. diluviana*, ought to be reinvestigated in light of the present results. Jokela-Määttä et al. (2005) reported a wide variation range (495–529 nm) of single-rhabdom *λ*_max_ measured in four individuals from two lakes in Idaho, USA (Hayden Lake and Pend Oreille). Cronin (2006) has published two within-individual average spectra, also from Idaho *M. diluviana*, one well-fitted by a 505 nm A1 template, the other by a 517 nm A2 template. The common interpretation at the time was that this species uses A1 ↔ A2 chromophore exchange. Yet, as Cronin (2006) notes, the A2 pair of an A1 pigment peaking at 505 nm should have *λ*_max_ = 535 nm, so the broad 517 nm spectrum would have arisen from a mixture. In fact, it is quite similar to the spectra we have here obtained from the marine *M. mixta* (*λ*_max_ = 517–525 nm; Table 1). Gal et al. (1999) reported an A1 pigment in *M. diluviana* from Cayuga Lake (NY, USA) with *λ*_max_ at 520 nm, and together with the 505 nm pigment, this might obviously produce a 517 nm A1/A1 mixture.

As argued above, it seems unlikely that *M. diluviana* should have an A1 ↔ A2 system that is absent in *M. relicta*. Rather, the variation of spectral sensitivity in *M. diluviana* might indicate differences in how the expression of the two opsins is regulated. It is interesting that both Cronin (2006) and Gal et al. (1999) were able to record seemingly pure A1 spectra from whole rhabdoms, suggesting that *M. diluviana* expresses either of its two opsins alone at least in some rhabdoms. Obviously, it can also express mixtures, as shown by Cronin’s 517 nm spectrum and by the wide spectral variation reported by Jokela-Määttä et al. (2005). Detailed study of *M. diluviana* as a sibling species of *M. relicta*, *M. salemaai* and *M. segerstralei* would be of special interest quite irrespective of the large-scale opportunities offered by the wider genus *Mysis*.

## Abbreviations

A1: Retinal
A2: 3,4-Didehydroretinal
ERG: Electroretinography
GFRKD: Standard visual pigment template of Govardovskii et al. (2000)
HPLC: High performance liquid chromatography
MSP: Microspectrophotometry
MWS: Middle wavelength sensitive
*L*: Lake
*L*_p_: *M. relicta* population of Lake Pääjärvi
LWS: Long wavelength sensitive
*S*: Sea
*S*_p_: *M. relicta* population of Pojoviken Bay of the Baltic Sea
SNR: Signal-to-noise ratio
QC: Quantum catch
WMTL: Wavelength of maximal transmission of light
*λ*_max_: Wavelength of maximal absorbance/sensitivity
